# Simultaneous Measurement of Magnetic Field and Temperature Utilizing Magnetofluid-Coated SMF-UHCF-SMF Fiber Structure

**DOI:** 10.3390/ma15227966

**Published:** 2022-11-11

**Authors:** Ronghui Xu, Yipu Xue, Minmin Xue, Chengran Ke, Jingfu Ye, Ming Chen, Houquan Liu, Libo Yuan

**Affiliations:** 1 Photonics Research Center, School of Optoelectronic Engineering, Guilin University of Electronic Technology, Guilin 541004, China; 2 Guangxi Key Laboratory of Optoelectronic Information Processing, Guilin University of Electronics Technology, Guilin 541004, China

**Keywords:** dual-parameter optical fiber sensor, magnetofluid, SMF-UHCF-SMF fiber structure

## Abstract

We have proposed and experimentally demonstrated a dual-parameter optical fiber sensor for simultaneous measurement of magnetic field and temperature. The sensor is a magnetofluid-coated single-mode fiber (SMF)-U-shaped hollow-core fiber (UHCF)-single-mode fiber (SMF) (SMF-UHCF-SMF) fiber structure. Combined with the intermodal interference and the macro-bending loss of the U-shaped fiber structure, the U-shaped fiber sensor with different bend diameters was investigated. In our experiments, the transmission spectra of the sensor varied with magnetic field strength and temperature around the sensing structure, respectively. The dip wavelengths of the interference spectra of the proposed sensor exhibit red shifts with magnetic field strength and temperature, and the maximum sensitivity of magnetic field strength and temperature were 1.0898 nm/mT and 0.324 nm/°C, respectively.

## 1. Introduction

Magnetic field sensors have found numerous potential applications in aerospace, underwater military, power grid, geological exploration, medical instrument and precision measurement [1,2,3,4,5]. There are many conventional magnetic field sensors, such as those based on the Hall effect, those using magneto-transistors or those that are magneto-resistive, that have some disadvantages, such as complicated structure, expensive cost, difficulty withstanding harsh environment conditions, being not easy to multiplex and to monitor remotely and so on [6,7]. Compared to conventional magnetic field sensors, fiber-optic-based magnetic field sensors have many benefits such as compact size, immunity to electromagnetic interference, remote monitoring, multiplexing capability, high sensitivity and resolution, good insulation and so on [8,9]. Therefore, optical fiber magnetic field sensors have aroused more and more attention during the past four decades [10,11].

Magnetic fluid (MF), also known as magnetofluid or magnetic liquid, is a stable colloidal liquid consisting of magnetic nanoparticles (Fe_3_O_4_, CoFe_2_O_4_ or MnFe_2_O_4_) coated with surfactant (oleic acid) and dispersed in a suitable liquid carrier (water, organic solvent or oil) [12,13]. MF possesses a unique magnetic-field-dependent refractive index (RI) as an external magnetic field is applied. Thus, MF exhibits some abundant magneto-optical characteristics, such as the Faraday effect, the birefringence effect, dichroism and tunable refractive index (RI), and can be used to design various optical devices such as wavelength filters, magneto-optical modulators, optical switches, magnetic field sensors and so on [14,15].

In recent years, many optic fiber magnetic field sensors combined with MF have been reported, which have different structures or principles, such as those based on tapered fiber [16,17], microstructure fiber [18,19], D-shaped fiber [20,21] or those based on interference mechanism [22,23,24,25], surface plasmon resonance (SPR) [26,27,28], photonic band gap effect [29,30] and so on. In 2016, Zhengyong Li et al. [24] reported an ultrasensitive magnetic field sensor based on a compact in-fiber Mach-Zehnder interferometer (MZI) created in twin-core fiber. This sensor has an ultrahigh magnetic field sensitivity of 20.8 nm/mT from 5mT to 9.5 mT. In 2020, Qianyu Lin et al. [31] developed the half-side-gold-coated MSM structure magnetic field sensor, and the sensor magnetic field intensity is 10.08 nm/mT at the magnetic field range of 2–12 mT. These sensors have high sensitivity. Nevertheless, the properties of the MF are also sensitive to temperature. Therefore, several fiber-optic sensors have also been reported to achieve the simultaneous measurement of magnetic field and temperature in recent years. An effective and simple method is cascaded with a fiber Bragg grating (FBG) [32,33,34]. In 2016, Yaofei Chen et al. [32] realized a macro-bending SMF cascaded with a FBG structure with the sensitivities of 1.426 nm/mT and 0.329 nm/°C. The macro-bending SMF and FBG induce two types of dips in the transmission spectrum of the sensor, which can be realized in dual-parameter measurement by monitoring the wavelength shifts of the two types of dips. In 2022, Yuxiu Zhang et al. [35] proposed a nonadiabatic tapered microfiber cascaded with FBG structure, and this sensor obtained high sensitivity of magnetic field and temperature that are 1.159 nm/mT and −1.737 nm/°C, respectively. This sensor can achieve high sensitivity, but sensors based on microfiber-nanofiber may be fragile. In addition to the method of cascading FBGs, some special structures can also measure the magnetic field and temperature at the same time. A single-mode-D-shaped-single-mode fiber structure was proposed by Yue Dong et al. in 2018 [36]. The D-shaped fiber is a side-polished fiber structure, and the fabrication of the sensor structure is time-consuming. The magnetic field intensity and temperature of this sensor are 0.997 nm/mT and 0.0775 nm/°C, respectively, and this sensor has high magnetic field sensitivity. In 2022, Dongying Wang et al. [37] designed a two-channel photonic crystal fiber (PCF)-SPR structure applied in detecting the magnetic field and temperature. It requires gold film or other special materials in the film-plating process in different channels and is filled with polydimethylsiloxane (PDMS) in channel 2 to achieve a response to the temperature. This sensor’s maximum sensitivities of magnetic field intensity and temperature are 0.65 nm/mT and 0.52 nm/°C. In the same year, Bing Sun et al. [22] reported a SMF-(Mn_3_O_4_-PDMS-Air) FP cavity-SMF structure that detected magnetic field intensity and temperature, and this sensor has a high sensitivity of temperatures up to 1.16 nm/°C, and the maximum sensitivity of magnetic field intensity is 0.563 nm/mT. This sensor mainly focused on the measurement of small magnetic fields, and the fabrication methods of the sensor are relatively complex.

In this work, we propose a dual-parameter fiber sensor based on HCF and MF for simultaneously measuring magnetic field intensity and temperature. The proposed sensor probe consists mainly of a SMF-UHCF-SMF structure, with a UHCF sandwiched between two SMFs. The UHCF section is totally sealed in a U-shaped vessel filled with MF, with UV glue at both ends preventing the fluid from leaking out. In our experiment, the maximum magnetic field intensity sensitivity of 1.0898 nm/mT and temperature sensitivity of 0.324 nm/°C were obtained, respectively. Unlike other dual parameter sensors, our sensor is very simple to fabricate because only fusion splicing and encapsulation are involved. Our designed sensing structure has good sensitivity, good linearity of response, low cost, no additional FBG and good mechanical performance.

## 2. Preparation and Principle

**Figure 1:**
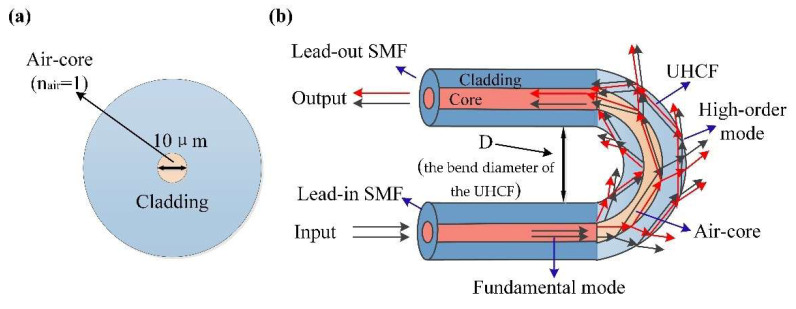
(**a**) Cross-section of the HCF. (**b**) Schematic diagram of the SMF-UHCF-SMF structure with optical path.

The HCF in the experiment is silica capillary with an inner diameter of 10 µm and a cladding diameter is 125 µm. The cross-section and parameters of the HCF are shown in Figure 1a. The SMF-UHCF-SMF structure is fabricated by sandwiching a length of HCF between two segments of SMFs. The fabrication process of the SMF-UHCF-SMF fiber structure is as follows:

Firstly, a segment of the HCF without a coating layer is spliced with a section of SMF with the coated layer stripped. We must ensure that the length of the HCF is 5 mm by fixed length cutting, and then another section of SMF with coated layer stripped is spliced at the other end of the HCF to form the SMF-HCF-SMF structure. Then, a regular U-shape vessel (the diameter of the vessel is 10 mm) is used as a simple bending guide, and the straight SMF-HCF-SMF fiber structure is bent to the SMF-UHCF-SMF structure. Finally, the SMF-UHCF-SMF structure is in a U-shaped vessel, and the vessel is filled with MF. To keep the optical fiber stable, both ends of the U-shaped vessel are sealed with UV glue.

Two sections of SMFs act as beam splitter and optical coupler, respectively. The high-order modes are excited at the spliced region of the lead-in SMF and the UHCF. The optical path of SMF-UHCF-SMF structure is shown in Figure 1b. When the lightwave guided in the lead-in SMF core is transmitted to the UHCF, due to the collapsed splicing region between the SMF and the UHCF, the incident lightwave diffracts and spreads out into the collapsed splicing region and excites high-order cladding modes. The high-order cladding modes propagate along the capillary wall and the air-core of the UHCF. In addition, there is a portion of incident lightwave that still propagates within the air-core of the UHCF and enters the capillary wall of the UHCF. When light in bent fibers leaks from the fiber core to the cladding, it causes a portion of ray radiation to penetrate the fibers and reflect onto the interface between the fiber cladding and the air [38]. At the spliced region of the lead-in SMF and the UHCF, the fundamental mode in the lead-in SMF is split into the air-core modes and the high-order cladding modes. The cladding modes and the air-core mode propagate along the UHCF. These mixing modes experienced different bending attenuation; meanwhile, they have different responses to the environmental RI (ERI). The air-core modes and the cladding modes mutually interfere at the splicing region of the lead-out SMF and the UHCF. The interference lightwave of the sensor is led-out from the lead-out SMF and observed by an OSA. In addition, the losses mainly happen where a fiber’s straight section transitions to a curved section (i.e., where the bend radius changes) [39]. Through conformal transformation, the SMF-UHCF-SMF structure with bending diameter D can be regarded as an equivalent straight waveguide (ESW). The effective refractive index (ERI) of ESW can be expressed as:

(1) $$n_{esw}=n(x,y)(1+\frac{x}{D})$$

where x is the diameter of the fiber, and D is the bend diameter of the UHCF. In addition, n(x,y) is the original RI of the straight fiber and $n_{esw}$ is the RI of the outer portion of the fiber. At the lead-out SMF, the cladding modes and the core mode couple back to the SMF core; thus, the multiple-mode interference effect will produce. According to the interference theory, the transmission spectra of the SMF-UHCF-SMF structure can be described as [40,41,42]:

(2) $$I_{out}=I_{core}+I_{clad}+2\sqrt{I_{core}I_{clad}}\cos\phi$$

where $I_{out}$ is the output light intensity, and $I_{core}$ and $I_{clad}$ represent the light intensity of fundamental mode and the cladding modes of the HCF participating in the interference, respectively. In addition, ϕ is the phase difference between the fundamental mode and the cladding modes, which can be expressed as follows:

(3) $$\phi =\frac{\left(n_{core}^{eff}-n_{clad}^{eff}\right)}{\lambda }L$$

λ is the wavelength of the incident light, L is the length of the UHCF and $n_{core}^{eff}$ and $n_{clad}^{eff}$ are the effective RIs of the core mode and the cladding mode, respectively. Then the interference spectrum shift wavelength is given by:

(4) $$\lambda_{dip}=\frac{2}{2m+1}\Delta n_{eff}L$$

where $\Delta n_{eff}$ is the ERI difference between the core mode and the cladding mode, $\Delta n_{eff}=n_{core}^{eff}-n_{clad}^{eff}$, because of the change in the RI of the external environment, so from Equation (4), it can be reasoned that the RI changes in the ambient environment will affect the resonance wavelength of the dip.

According to Equation (4), we can calculate that the position of the interference dip depends on the effective refractive index difference between the fundamental mode and the cladding mode when the interference path length is fixed. The effective RI of the cladding mode changes with the external environment RI, while the effective RI of the core mode does not change, which makes the position of the interference dip change. Therefore, the external environment variables can be detected and measured by monitoring the changes of the wavelength of the interference dip. Compared to the SMF-HCF-SMF structure, the SMF-UHCF-SMF structure has a bending oscillation interference and intermodal interference form similar to the MZI effect.

The RI of magnetic fluid is affected not only by magnetic field intensity but also temperature, and the relationship can be described as [43]:

(5) $$n\left(H\right)=\left\{ \begin{array}{c} n_{0},H\leq H_{c}\\ n_{s}-n_{0}\left[\coth\left(\frac{H-H_{c,n}}{T}\right)-\frac{T}{\alpha \left(H-H_{c,n}\right)}\right]+n_{0},H>H_{c,n} \end{array}\right.$$

where $H_{c,n}$ is the critical magnetic field intensity, $n_{s}$ is the saturation RI of MF, $n_{0}$ is the RI when the magnetic field intensity is less than $H_{c,n}$, α is the fitting parameter and H and T are the external magnetic field intensity and temperature, respectively.

## 3. Experimental Results and Discussion

**Figure 2:**
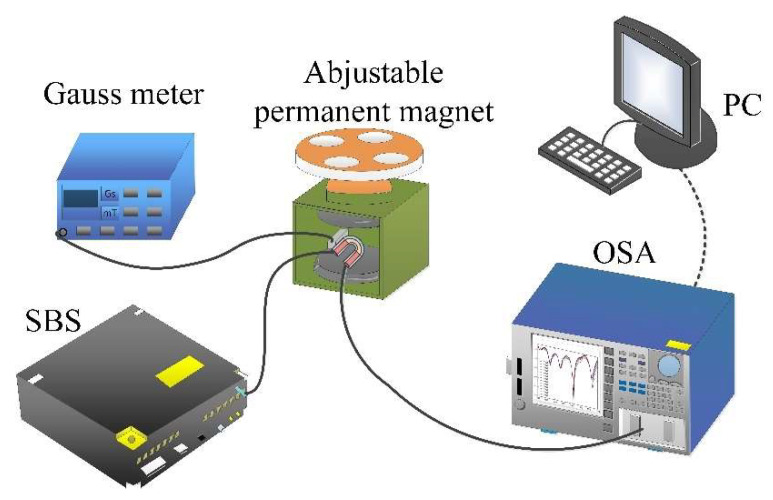
Experimental setup of the magnetic field measurement.

The experimental setup of the magnetic field measurement is illustrated in Figure 2. In our experiment, the SMF-UHCF-SMF structure or the sensor head was fixed in the center of an adjustable permanent magnet (YP Magnetic Technology Development Co., Changchu, China), which was used to supply a uniform magnetic field source. The magnetic field intensity of the magnetic field source was monitored by a Gauss meter with resolution of 0.1 mT. The continuous light from the supercontinuum broadband light source (SBS, Wuhan Yangtze Soton Laser Co., Ltd., Wuhan, China) was emitted into the magnetic field sensor by the lead-in SMF. The SBS wavelength range was 600 nm–1700 nm. The transmission spectrum of the sensor was outputted from the lead-out SMF and observed by an optical spectrum analyzer (OSA, Yokogawa, AQ6370C, Tokyo, Japan) with a spectral resolution of 0.02 nm.

### 3.1. Refractive Index Measurement

Since the prepared sensor is based on the adjustable RI feature of the MF to measure magnetic field intensity, we needed to explore the RI sensitivity of the sensor firstly. In order to select an optimal bending curvature and prevent the sensing structure (SMF-UHCF-SMF structure) to break, the bending diameter of the UHCF could not be selected too short. Additionally, the bending diameter of the UHCF could not be too large for obtaining a high sensitivity. Thus, we chose three sensor samples with bend diameter of the UHCF around 47.0 mm, 50.0 mm and 54.0 mm, respectively, to verify and choose an optimize sensing structure.

**Figure 3:**
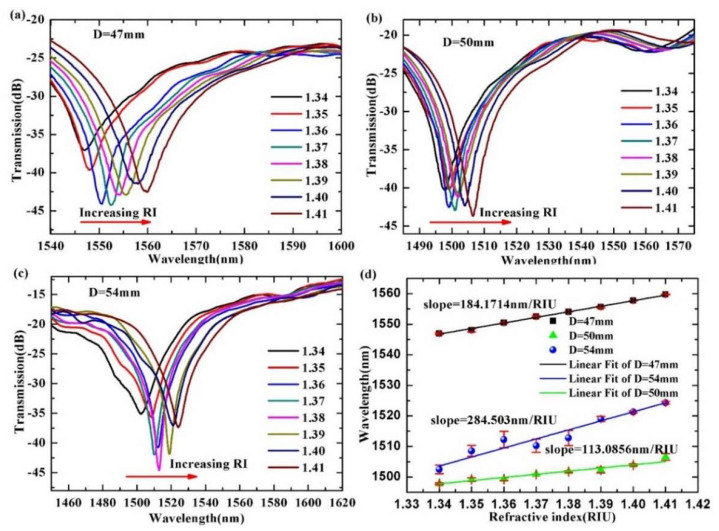
Transmission spectra and the wavelength shift trend of the corresponding valley of the SMF-UHCF-SMF structure immersed in solution with different RIs. (**a**) the bending diameter of the UHCF is 47mm; (**b**) the bending diameter of the UHCF is 50mm; (**c**)the bending diameter of the UHCF is 47mm; (**d**) the wavelength shift trend of the corresponding valley with different RIs.

When the room temperature was maintained at 25 °C, the response of the sensor to external RI was studied by completely immersing the sensor in a glycerol solution. RI solutions consisted of glycerol mixed with water in various proportions. The RI of the solution was calibrated by an Abbe refractometer. The actually measured corresponding RIs were 1.340, 1.350, 1.360, 1.370, 1.380, 1.390, 1.400 and 1.410, respectively. The transmission spectra and the wavelength shift of three sensor samples in the different RI are shown in Figure 3.

Figure 3a–c show the transmission spectra of the three selected sensing structure parameters or the bending diameter of the HCF with the refractive index. From Figure 3a–c, we can see the changes of the three sensor samples in transmission spectra are similar. The interference spectra shift toward longer wavelength (red shift), and the transmission loss also change as the glycerol RI increases. In the range of 1500~1600 nm, the fitting results show that the RI shift is linear, the RI sensitivity of D = 47 mm is 184.17143 nm/RIU, the RI sensitivity of D = 50 mm is 113.0856 nm/RIU and the RI sensitivity of D = 54 mm is 284.503 nm/RIU. To represent the accuracy of the data, we added error bar analysis to the discrete data. As shown in Figure 3d, these red vertical lines represent data errors, and the error bars (red vertical lines) indicate that the RI sensing error is little.

Actually, in the experimental results, the sensing structure with 50 mm exhibits lower RI sensitivity than that of ones with 47 and 54 mm. The RI sensitivity does not show an obviously regularity with the diameter or curvature of the UHCF among the three sensing samples. This may be related to the different high-order cladding modes involved in the intermodal interference.

**Figure 4:**
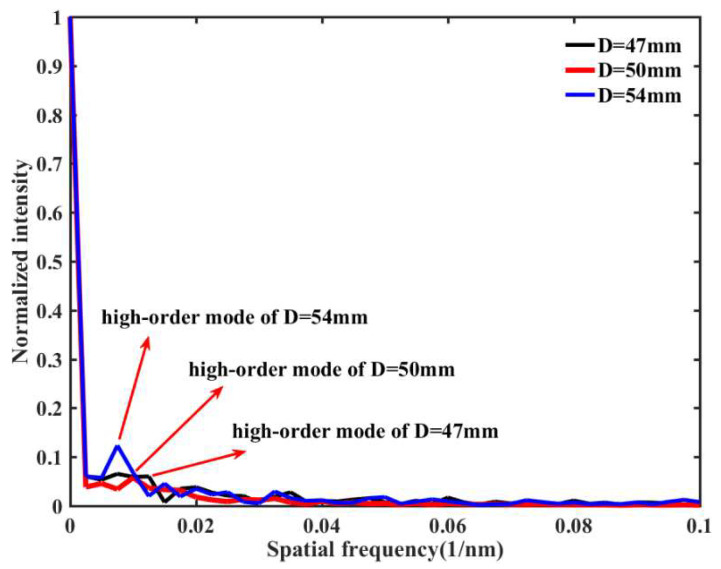
Spatial frequency distribution of transmission spectra of the proposed sensor samples with different bending diameter (D = 47.0 mm, 50.0 mm, 54.0 mm).

Figure 4 shows the spatial frequency spectra of three different transmission spectra, which corresponds to different sensor bending diameter (D = 47.0 mm, 50.0 mm, 54.0 mm). Figure 4 shows the high-order modes involved in the intermodal interference for the interference valleys of the three sensor samples. We believe that the sensing structures with different bending diameters excite different high-order cladding modes. In addition, due to these different high-order modes, we achieve individual transmission attenuation. Moreover, these high-order modes have different sensitivities to the ERI. Because the SMF-UHCF-SMF structure with a bending diameter of 54 mm has higher RI sensitivity, we choose this bending diameter as the sensor structure parameter.

### 3.2. Magnetic Field Intensity Measurement

**Figure 5:**
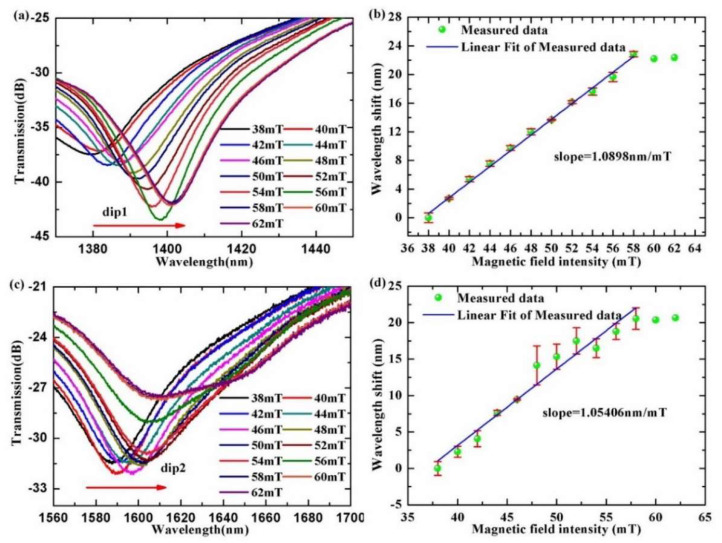
(**a**,**c**) Wavelength shifts of the dip1 and the dip2 of the interference spectra with the magnetic field intensity. (**b**,**d**) The wavelength shift trends of the dip1 and the dip2 of the interference spectra with magnetic field intensity.

To investigate the transmission spectrum of the SMF-UHCF-SMF sensor structure to magnetic field intensity, we kept the sensor structure in the center of the adjustable permanent magnet and then gradually changed the intensity of the magnetic field by adjusting the distance between the poles. In addition, the magnetic field direction was perpendicular to the position of the U-shaped surface of the SMF-UHCF-SMF structure. From Figure 5, one can find that the transmission spectrum has two adjacent interference dips (the dip1 and the dip2), and the transmission spectra shift toward a long wavelength (or red shift) in the measurement range from 38 mT to 62 mT.

From Figure 5a, one can find that the wavelength of the dip1 shifts monotonically from 1378.48 nm to 1401.33 nm with magnetic field intensity. Similarly, we can observe that the wavelength of the dip2 shifts monotonically from 1587.19 nm to 1607.76 nm in Figure 5c. Additionally, when the magnetic field intensity increases to 58 mT or a larger value, the wavelength shifts of the transmission spectra are hardly varied. We understand that the saturation magnetic field strength of the magnetic fluid is reached. The two dips in the transmission spectrum of the sensing structure correspond different interference modes. The different modes propagate in the sensing structure experience different loss. Thus, different interference modes exhibit different response to the magnetic field intensity or the RI of the magnetic field around the sensing structure. The wavelength sensitivity to magnetic field of the dip1 and the dip2 are shown in Figure 5b,d, respectively. The fitting results in Figure 5b indicate that the response of the absolute wavelength shift as a function of magnetic field intensity is generally linear. Furthermore, the magnetic field intensity response has good linearity (R^2^ = 0.99) from 38 mT to 58 mT. The maximum sensitivity of wavelength shift is 1.0898 nm/mT. Figure 5d shows that maximum wavelength sensitivity to magnetic field of the dip2 is 1.05406 nm/mT, and the linearity is 0.95 in the same measurement range of magnetic field intensity. Similarly, we added error bars (red vertical lines) to represent the accuracy of the data, and the error bars (red vertical lines) indicate that the magnetic field sensing error is little.

### 3.3. Temperature Measurement

**Figure 6:**
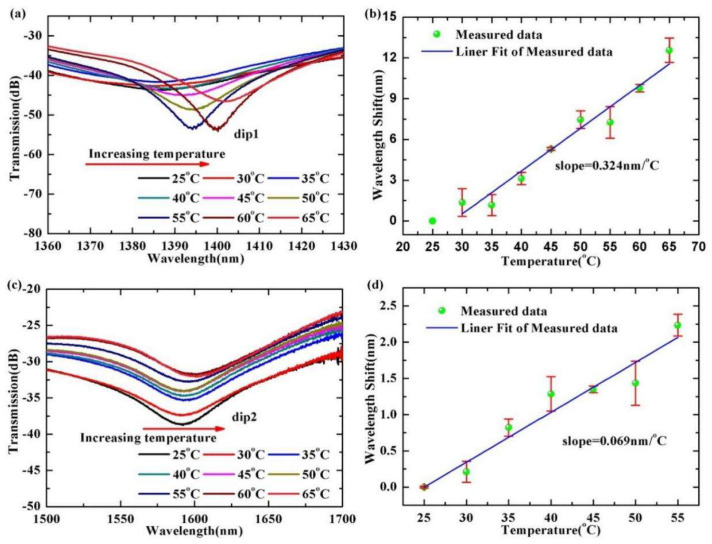
(**a**,**c**) Wavelength shifts of the dip1 and the dip2 of the interference spectra with the temperature. (**b**,**d**) The wavelength shift trends of the dip1 and the dip2 of the interference spectra with temperature.

In order to investigate the temperature sensitivity of the sensor, we measured different temperatures and monitored wavelength shift of the dips of the transmission spectra by the OSA. The sensor was placed on a heating platform to ensure that the optical fiber structure was not stretched by external axial tension. We placed a thermometer probe near the sensing structure to monitor the temperature. We measured temperature in steps of 5 °C, within the temperature range of 25 °C to 65 °C. After reaching the set temperature, it remained steady for 20 min per step to reduce any measurement error. Figure 6a,c show the wavelength shift of the dip1 and the dip2 under the range of 25 °C to 65 °C. The data fitting results in Figure 6b,d indicate that the response of the absolute wavelength shift as a function of temperature is generally linear. Temperature sensitivities of the dip1 and the dip2 are 0.324 nm/°C and 0.069 nm/°C, respectively. The error bars (red vertical lines) in the fitted lines imply that the errors are very low.

### 3.4. Analysis of Experimental Results

According to the above experimental results, we ascertained that the interference of different order modes leads to the difference of magnetic field strength and temperature sensitivity. By monitoring the wavelength shifts of the dip1 and the dip2 of the transmission spectra, magnetic field intensity and temperature can be measured simultaneously. The relationship between wavelength shift and magnetic field intensity and the relationship between wavelength shift and temperature are described in the following equation:

(6) $$\left[\begin{array}{c} \Delta \lambda_{dip1}\\ \Delta \lambda_{dip2} \end{array}\right]=\left[\begin{array}{cc} K_{H,dip1} & K_{T,dip1}\\ K_{H,dip2} & K_{T,dip2} \end{array}\right]\left[\begin{array}{c} \Delta H\\ \Delta T \end{array}\right]$$

where $\Delta \lambda_{dip1}$ and $\Delta \lambda_{dip2}$ represent the changes of wavelength shifts at the dip1 and the dip2, respectively,$K_{H,dip1}$ and $K_{T,dip1}$ denote the sensitivities of the magnetic field intensity and temperature for the dip1. Similarly, $K_{H,dip2}$ and $K_{T,dip2}$ are those for the dip2. ΔH expresses the change of external magnetic field intensity, and ΔT is the temperature change around the sensor structure.

By taking the inverse matrix of the coefficient matrix in Equation (7), the changes in magnetic field intensity and temperature can be calculated from the wavelength shifts of the transmission spectra of the dip1 and the dip2 and are expressed as follows:

(7) $$\left[\begin{array}{c} \Delta H\\ \Delta T \end{array}\right]={\left[\begin{array}{cc} K_{H,dip1} & K_{T,dip1}\\ K_{H,dip2} & K_{T,dip2} \end{array}\right]}^{-1}\left[\begin{array}{c} \Delta \lambda_{dip1}\\ \Delta \lambda_{dip2} \end{array}\right]$$

Based on the above experimental results, $K_{H,dip1}$ = 1.0898, $K_{H,dip2}$ = 1.0506, $K_{T,dip1}$ = 0.324 and $K_{T,dip2}$ = 0.069. When we substitute these into Equation (7), we can achieve:

(8) $$\left[\begin{array}{c} \Delta H\\ \Delta T \end{array}\right]=-\frac{1}{0.2652}\left[\begin{array}{cc} 0.324 & -0.069\\ -1.0506 & 1.0898 \end{array}\right]\left[\begin{array}{c} \Delta \lambda_{dip1}\\ \Delta \lambda_{dip2} \end{array}\right]$$

With this matrix, the magnetic field intensity and temperature variation can be calculated out after measuring the resonant wavelength shifts of the dip1, and the dip2.

### 3.5. Comparison with Other Sensing Structure

In comparison with some other dual-parameter sensors of magnetic field and temperature, we list the reported works in Table 1. From the table, one can find easily that, all in all, the proposed dual-parameter fiber sensor has a good sensitivity. Additionally, compared with some reported works, such as those based on side-polished fiber structure, taped-fiber structure, MF-infiltrated PCF structure, gold film-coated fiber SPR structure, Mn_3_O_4_-PDMS cap structure, optical fiber cascaded with a FBG structure and so on, our designed sensing structure is simple, low-cost, easy to fabricate, requires no additional FBG and has good mechanical performance.

## 4. Conclusions

In conclusion, by using magnetofluid encapsulated a U-shaped HCF sandwiched between two segments of SMFs, we have achieved a dual-parameter optical fiber sensor of magnetic field and temperature. At the spliced region of the lead-in SMF and the UHCF, the fundamental mode in the lead-in SMF is split into the air-core modes and the high-order cladding modes. The cladding modes and the air-core mode propagate along the UHCF. These mixing modes experienced different bending attenuation; meanwhile, they have different responses to the environmental RI (ERI). The air-core modes and the cladding modes mutually interfere at the splicing region of the lead-out SMF and the UHCF. The interference lightwave of the sensor is led-out from the lead-out SMF and observed by an OSA. The experimental results show that interference dips of the transmission spectra are sensitive to magnetic field intensity and temperature around the sensor structure. The maximum magnetic field intensity and temperature sensitivities are 1.0898 nm/mT and 0.324 nm/°C, respectively. The proposed optical fiber magnetic field sensor has the advantages of simple structure, low cost, convenient to fabrication and good mechanical performance and may provide a potential candidate for industry applications.

## Figures and Tables

**Table 1:** Comparison with some other dual-parameter sensors of magnetic field and temperature.

Sensing Structure	Sensitivity	Ref.
SMF-(Mn_3_O_4_-PDMS-Air) FP cavity-SMF Structure	0.563 nm/mT (0–4 mT) 1.16 nm/°C (30–70 °C)	[22]
SMF-MF infiltrated PCF-SMF	0.72 nm/mT (0–6.66 mT); −0.08 nm/°C (20–60 °C)	[29]
Macro-bending SMF cascaded with a FBG	1.426 nm/mT (3–10 mT) 0.329 nm/°C (28.6–57.2 °C)	[32]
MF-infiltrated PCF Cascaded with a FBG	0.925 nm/mT (0–10 mT); 0.165 nm/°C (20–70 °C)	[33]
Two-taped Fiber Joints Cascaded with a FBG	0.408 nm/mT(0–25 mT); −0.363 nm/°C(25–55 °C)	[34]
A nonadiabatic tapered microfiber Cascaded with a FBG	1.159 nm/mT (0–18 mT) −1.737 nm/°C (25–50 °C)	[35]
SMF-D-shaped Fiber-SMF	0.997 nm/mT (0–2.1 mT); 0.0775 nm/°C (30–55 °C)	[36]
SMF-exposed core fiber (ECF)-SMF	−0.18 nm/mT (0–7.3 mT); −0.16 nm/°C (26–50 °C)	[44]
SMF-no-core fiber (NCF)-SMF	0.074 nm/mT (2–16 mT); −0.28667 nm/°C (20–70 °C)	[45]
Two-channel PCF-SPR Structure	0.65 nm/mT (5–13 mT); −0.52 nm/°C (17.5–27.5 °C)	[37]
SMF-UHCF-SMF	1.0898 nm/mT (38–62 mT); 0.324 nm/°C (25–65 °C)	This work

## Data Availability

The original contributions presented in the study are included in the article. Further inquiries can be directed to the corresponding author.
